# Densely Convolutional Spatial Attention Network for nuclei segmentation of histological images for computational pathology

**DOI:** 10.3389/fonc.2023.1009681

**Published:** 2023-05-25

**Authors:** Rashadul Islam Sumon, Subrata Bhattacharjee, Yeong-Byn Hwang, Hafizur Rahman, Hee-Cheol Kim, Wi-Sun Ryu, Dong Min Kim, Nam-Hoon Cho, Heung-Kook Choi

**Affiliations:** ^1^ Department of Digital Anti-Aging Healthcare, Ubiquitous-Anti-aging-Healthcare Research Center (u-AHRC), Inje University, Gimhae, Republic of Korea; ^2^ Department of Computer Engineering, Ubiquitous-Anti-aging-Healthcare Research Center (u-AHRC), Inje University, Gimhae, Republic of Korea; ^3^ Artificial Intelligence R&D Center, JLK Inc., Seoul, Republic of Korea; ^4^ Department of Pathology, Yonsei University Hospital, Seoul, Republic of Korea

**Keywords:** computational pathology, nuclei segmentation, histological image, attention mechanism, deep learning

## Abstract

**Introduction:**

Automatic nuclear segmentation in digital microscopic tissue images can aid pathologists to extract high-quality features for nuclear morphometrics and other analyses. However, image segmentation is a challenging task in medical image processing and analysis. This study aimed to develop a deep learning-based method for nuclei segmentation of histological images for computational pathology.

**Methods:**

The original U-Net model sometime has a caveat in exploring significant features. Herein, we present the Densely Convolutional Spatial Attention Network (DCSA-Net) model based on U-Net to perform the segmentation task. Furthermore, the developed model was tested on external multi-tissue dataset – MoNuSeg. To develop deep learning algorithms for well-segmenting nuclei, a large quantity of data are mandatory, which is expensive and less feasible. We collected hematoxylin and eosin–stained image data sets from two hospitals to train the model with a variety of nuclear appearances. Because of the limited number of annotated pathology images, we introduced a small publicly accessible data set of prostate cancer (PCa) with more than 16,000 labeled nuclei. Nevertheless, to construct our proposed model, we developed the DCSA module, an attention mechanism for capturing useful information from raw images. We also used several other artificial intelligence-based segmentation methods and tools to compare their results to our proposed technique.

**Results:**

To prioritize the performance of nuclei segmentation, we evaluated the model’s outputs based on the Accuracy, Dice coefficient (DC), and Jaccard coefficient (JC) scores. The proposed technique outperformed the other methods and achieved superior nuclei segmentation with accuracy, DC, and JC of 96.4% (95% confidence interval [CI]: 96.2 – 96.6), 81.8 (95% CI: 80.8 – 83.0), and 69.3 (95% CI: 68.2 – 70.0), respectively, on the internal test data set.

**Conclusion:**

Our proposed method demonstrates superior performance in segmenting cell nuclei of histological images from internal and external datasets, and outperforms many standard segmentation algorithms used for comparative analysis.

## 1 Introduction

The segmentation of cell nuclei from a histopathological image has been a focus of clinical practice and scientific research for more than half a century (1). Histopathology images obtained from a biopsy may help to determine the stage of cancer, based on the morphology of cell nuclei, and provide critical clues to healthcare providers (2). Histopathological images permit early detection of tumors, but manually analyzing these images is difficult. Current developments in digital pathology have the potential to reduce the workload of pathologists by overcoming the mass segmentation and low inter-rater agreement (3). Image segmentation is typically used to discover objects and boundaries (lines, curves, etc.) in images and distinguish between foreground and background. The purpose of segmentation is to simplify or change the visualization of an image into something that is more meaningful and easier to analyze.

Manually recognizing and annotating medical images is a time- and labor-intensive task. Research into computer-aided medical image segmentation has flourished in recent years; this is a great benefit of the expanding collaboration between artificial intelligence and medical image analysis. Computer-assisted segmentation allows clinicians to quickly and easily create image markers relevant to the illness treatment process, which allows them to discover malignant tissue affected in an early stage. Pathologists can swiftly extract significant morphological features from the histological image, in particular with automatic segmentation of images of tissue stained with hematoxylin and eosin (H&E). This approach enables pathologists to serve a larger number of patients while maintaining diagnostic accuracy. It can, to some extent, alleviate the problems of unequal distribution of medical resources and a scarcity of skilled pathologists. In addition, nuclei segmentation can yield information about the shape of the gland, which is important for grading cancer (4).

Nuclear segmentation provides both logical and pivotal starting points for histopathological image analysis, feature extraction, detection, and classification *via* computer algorithms. However, accurate segmentation is important for identifying abnormalities in the histological sections. In addition, it is a challenging task to segment the nuclei in an H&E-stained image because of chromatic stain variability, nuclear overlap and occlusion, variability in optical image quality, stain density, and differences in nuclear and cytoplasmic morphology (5–8). The distribution and shape of cell nuclei in the histological sections determine the stage of cancer and the prognosis (9). Moreover, layers of epithelium on the interior and exterior of an organ can be identified from nuclear presence or absence, morphology, and distribution (5). Traditional automatic nuclei segmentation approaches (e.g., clustering, intensity thresholding, active contour, region growing, level set) fail with noisy images and clumped nuclei and are computationally expensive (7, 8, 10, 13, 14). Therefore, these techniques are not very robust compared to machine learning, in particular a convolutional neural network (CNN), which can learn to identify variations in nuclear morphology and staining patterns (8, 15, 16). Deep CNN has received great attention and is the dominant technique for biological object detection and segmentation in medical imaging (17–19). Deep-learning (DL) algorithms assist pathologists in interpreting whole-slide images (WSIs) to detect tumor regions (20, 21). The major challenge of nuclei segmentation is arranging the annotation data, which is required for neural network models that involve training. However, nuclei segmentation data sets consisting of H&E-stained images from multiple organs with nuclear annotations are publicly available online. The data sets are released for research and participation in segmentation challenges (15, 16, 22–27).

In this paper, we propose a CNN model named Densely Convolutional Spatial Attention Network (DCSA-Net) based on U-Net to perform the nuclei segmentation of histological images of PCa. Our primary goal is to accurately segment the cell nuclei in images of PCa tissue and validate our model on the public dataset – MoNuSeg, which includes liver, breast, kidney, bladder, prostate, and stomach images. In addition, we are releasing the source code of our proposed model to aid in its use, evaluation, and improvement. In the proposed model, two attention modules are connected to enhance local related features and reuse them at spatial and channel levels. Inspired by the existing work, we have also introduced and released a data set that contains 75 PCa pathology images of size 512 × 512 pixels, with more than 16,000 hand-annotated nuclei, sourced from two different hospitals. The PCa images were annotated using Apeer Web Application tool and we ensure that the nuclei were correctly annotated. In the remainder of this paper, we briefly review the research on nuclei segmentation, then describe the data set arrangement and its pre- and post-processing along with details of the proposed model. The output results of various CNN models are then compared with our proposed DCSA-Net. Finally, we discuss the results of nuclei segmentation and present our conclusions.

## 2 Related work

Depending on the image intensity, each pixel is classified as a nucleus or background. This method is susceptible to disturbances, uneven backgrounds, and intensity heterogeneity within the images, yet it delivers effective segmentation performance for tissue images that contain uniform backgrounds. For example, the size, shape, and texture of cell nuclei differ in images of the breast and cervical tissue. Nuclei segmentation in cytology and histology sections frequently revolves around thresholding, clustering, and active contouring. However, because of a variety of nuclear appearances in the histological sections, traditional methods do not perform equally well for all kinds of tissue images.

Traditional image segmentation algorithms work by dividing an image into sections that contain comparable features, such as color and texture (28). Wu et al. (29) presented a region-growing algorithm for the segmentation of human intestinal glands. Their method performs well for most images of normal and abnormal intestinal glands, and the segmentation results are sensitive to both the number of clusters and region initialization. Bhattacharjee et al. (30) used K-means and watershed algorithms, respectively, to segment tissue components and separate overlapping cell nuclei. This method performs well for images of PCa tissue that exhibit Gleason pattern (GP) 3 and 4 but not GP 5 because of the high abnormality and heterogeneity of the cell nuclei. Yi et al. (31) proposed an automated approach to cell nuclei segmentation that works with H&E-stained images. They used a color deconvolution algorithm to get the hematoxylin channel and used a morphological operation and thresholding technique to detect nuclei and background regions. Moreover, the detected regions were used as markers for a marker-controlled watershed segmentation algorithm. The proposed method shows promising results in terms of segmentation accuracy and separation of touching nuclei.

In recent years, DL algorithms have become more popular than traditional methods of cell nuclei segmentation. Many researchers have used different CNN-based models that can automatically learn advanced features from the image for classification, detection, and segmentation. Long et al. (32) performed semantic segmentation using the classification networks AlexNet, VGGNet, and GoogleNet by transferring their learned representations and fine-tuning them. These were the first methods to be used for image-semantic segmentation using end-to-end deep neural networks. The methods of medical image segmentation have progressed from manual to semi-automated and finally to fully automatic segmentation (33, 34). Ronneberger et al. (35) proposed a network (i.e., U-Net) for microscopy image segmentation and won the International Symposium on Biomedical Imaging Challenge in 2015. Their architecture achieved promising performance on a variety of biomedical segmentation applications. Several modifications have since been suggested in the architecture of U-Net to improve its accuracy. Badrinarayanan et al. (36) presented a novel CNN architecture for semantic pixel-wise segmentation called SegNet. In this architecture, the encoder network is topologically identical to the VGG-16 layers (37). SegNet is designed to be efficient in terms of both memory and computational cost during inference. Similarly, different CNN models have been proposed as the backbone (i.e., encoder network) of U-Net and LinkNet (38, 39) for semantic segmentation. Furthermore, the U-Net architecture has been improved in many ways to give rise to Residual-UNet (40), Dense-UNet (41), Inception-UNet (42), and so forth. Among the many networks developed, multi-scale and stacked networks have garnered great attention from the research community. The presence of skip connection based on concatenation or addition functions in residual, dense, and inception units makes information flow easier for the entire network, alleviates the vanishing-gradient problem, strengthens feature propagation, encourages feature reuse, and substantially reduces the number of parameters.

In another bid to improve the performance of semantic segmentation, researchers have developed an attention unit that can be used as a plug-and-play module in the existing CNN architecture. With the help of this attention module, the model automatically focuses to learn the target structures of varying shapes and sizes. Oktay et al. (43) proposed a network called Attention U-Net for medical imaging that is controlled by an attention gate (AG). Because of the AGs, the prediction performance of U-Net consistently improved while computational efficiency was preserved. Cheng et al. (44) proposed an attention block that can capture feature dependencies in channel and space dimensions. Using this block, they obtained a new ResNet variant called ResGANet. Its success with a variety of medical image classification tasks shows that the proposed ResGANet is superior to the backbone model. He et al. (45) proposed a hybrid-attention nested U-Net for nuclei segmentation. The model contains two modules: a hybrid nested U-shaped network and an attention block. Their model extracts feature that effectively segment the boundaries of diverse, small, and dense nuclei. Zhao et al. (46) introduced a semantic segmentation network called Spatial-Channel Attention U-Net (SCAU-Net) based on the current research status of medical images. The key point of this model is to enhance local features and restrain irrelevant details at the spatial and channel layers. They performed experiments on the gland data set, and the model showed superior results in an image segmentation task compared to the classic U-Net. The stardist model (47) was proposed to localize cell nuclei *via* star-convex polygon performed well as compared to other models with better shape representation and thus do not need shape refinement.

## 3 Materials and methods

**Figure 1:**
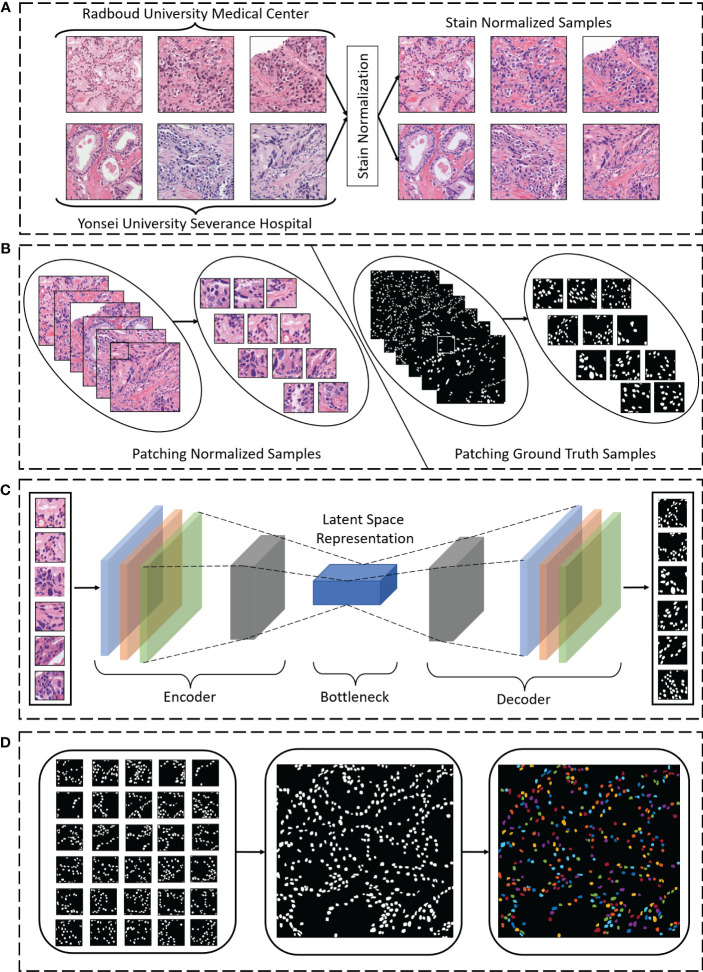
**(A)** Hematoxylin and eosin–stained samples from Radboud University Medical Center and Yonsei University Severance Hospital were stain normalized during pre-processing of the images. **(B)** Normalized and ground truth sample images were broken up into smaller sizes (“patches”) during pre-processing. **(C)** Nuclei segmentation using the proposed encoding and decoding technique was performed. **(D)** During post-processing, the segmentation patches were merged to reconstruct the original shape, and the cell nuclei were color-mapped for visualization.

The workflow of nuclei segmentation was divided into three phases, as shown in **Figure 1**: pre-processing, segmentation, and post-processing. First, we pre-processed raw image patches (512 × 512 pixels) using the stain normalization technique (**Figure 1A**). Then we broke the patch images up into small sizes (128 × 128 pixels), to carry out segmentation more efficiently (**Figure 1B**). Next, we used our proposed state-of-the-art model for nuclei segmentation and compared our results to those of other methods (**Figure 1C**). Finally, we post-processed the segmented patch images to reconstruct the original shape for better visualization of the segmentation (**Figure 1D**).

### 3.1 Dataset

Several nuclei segmentation data sets with complete nuclei annotation and multi-organ histopathology images are publicly available. However, in this study, to perform the nuclei segmentation, we cropped sub-images of size 512 × 512 pixels from both Radboud University Medical Center (RUMC) and Yonsei University Severance Hospital (YUHS) PCa WSIs and selected 75 samples (45 from RUMC and 30 from YUHS) for nuclei annotation. However, the dataset of 75 samples was created using 10 different patients (RUMC - 6 and YUHS - 4). After obtaining 512 × 512 sub-images, we annotated more than 16,000 nuclei using the Apeer Web Application, which is publicly accessible at https://www.apeer.com/home/. The annotators were engineering students working as research assistants and were trained by co-authors to identify nuclei in histological sections of PCa. Nonetheless, the sub-images were uploaded to the Apeer Web Platform to perform nuclei annotation. The annotations included only foreground pixels (i.e., cell nuclei), and overlapping nuclei are separated into multi-nuclear pixels for better segmentation. Furthermore, the quality of annotated images was evaluated by the co-authors before preprocessing and training steps. The generated annotation files are binary images containing 0 and 255 intensities. **Supplementary Figure S1** shows a few example samples from RUMC and YUHS datasets. A total of 3,675 patches (2205 from RUMC and 1470 from YUHS) of size 128 × 128 pixels from 75 images (512 × 512 pixels) were extracted to carry out patch-based nuclei segmentation. A detailed explanation of RUMC and YUHS datasets can be found in **Supplementary Material**.

A public dataset, Multi-organ Nucleus Segmentation (MoNuSeg), was obtained for external validation which is publicly available at https://monuseg.grand-challenge.org/Data/ (accessed on September 15, 2021). Kumar et al. (16) were the first to use the MonuSeg data set for generalized nuclear segmentation for computational pathology. They downloaded WSIs of digitized tissue samples of seven different organs, namely the liver, breast, kidney, bladder, prostate, colon, and stomach from 30 different patients. After obtaining 1000 × 1000 sub-images, they annotated more than 21,000 nuclear boundaries in Aperio ImageScope. However, to perform external validation, we included 6 images of size 512 × 512 pixels in our experiment. **Supplementary Figure S2** shows six different tissue samples from the MonuSeg dataset.

### 3.2 Stain normalization

In tissue engineering, stain normalization is an important part of pre-processing before the analysis is performed. Because of differences in image acquisition, tissue processing, staining protocols, and the response function of digital scanners, histopathological images vary greatly (e.g., in illumination, color, and quality of stain) (48). This variation is a major issue for CNN-based computational pathology methods. Several CNN models with different mechanisms perform detection, classification, and segmentation based on color and texture. In the case of tumor segmentation, if stain normalization is performed at the pre-processing step, the CNN algorithm demonstrates stable performance (49). Vahadane et al. (50) proposed a color normalization technique that preserves the structure in the source image while adapting the color to the target domain. This approach is very important for subsequent image analysis. Their method showed superior performance of stain normalization, validated both qualitatively and quantitatively. However, for our nuclei segmentation, we used a stain normalization method proposed by (51), which provides two mechanisms for overcoming many of the known inconsistencies in the staining process, thereby improving quantitative analysis.

### 3.3 Patch extraction and merging

To perform CNN-based nuclei segmentation, a patch generation method was developed to accurately segment the cell nuclei in a microscopic biopsy image. After the images were stain normalized, patches with a target size of 128 × 128 pixels were generated from a single image of size 512 × 512 pixels. From the top left corner of the image (px, py), shifting of the sliding window (ix, jy) from left to right and top to bottom was performed with grid spacing ix = jy = 64 along both row and column. This shifting method is shown in **Supplementary Figure S3A**. After training the model with the patch images, we merged the predicted segmented patches to reconstruct the original shape (i.e., 512 × 512 pixels), as shown in **Supplementary Figure S3B**.

### 3.4 Network architecture

**Figure 2:**
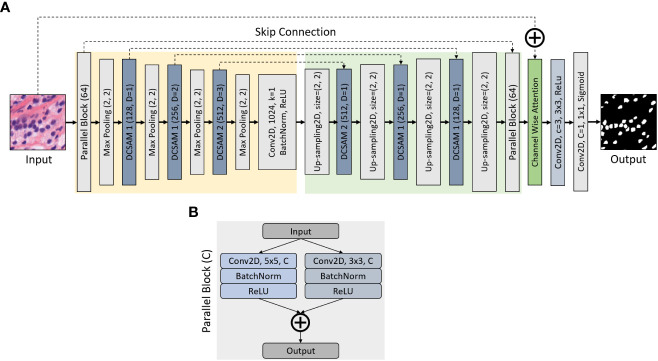
Structure of Densely Convolutional Spatial Attention Network. The entire structure is divided into four parts: **(A)** encoder, decoder, Densely Convolutional Spatial Attention Module, and **(B)** parallel convolutional blocks. Given an input feature map of size D × H × W, the output size is H × W × C, where W is the width, H is the height of the feature map, D is the input channel number, and C is the output channel number. The skip connection links the corresponding down- and up-sampling feature maps.

In this study, we propose a segmentation model (**Figure 2A**) inspired by the original U-Net (35), where we define a parallel block, which executes 5 × 5 and 3 × 3 2D convolutions in a parallel manner, followed by batch normalization and activation of a rectified linear unit (ReLU). The output of the parallel block (**Figure 2B**) is computed by concatenating two convolutional layers. Nonetheless, the purpose of using parallel-block in this network is to extract more diverse features from raw inputs. To improve the segmentation results, we introduce this parallel convolutional block, which is included in the encoder and decoder part of the network.

The entire architecture combines an encoding path with traditional convolutions (marked in yellow) and a decoding path with 2D-Upsampling (marked in green) to extract multilevel features from the input image. A segmented map is executed by gradually restoring the details and spatial dimensions of the image according to the learning features. In the encoder block, the image dimension is reduced because of the 2 × 2 max-pooling operation, while the number of feature channels is doubled during each down-sampling. The decoder is the opposite of the encoder block, where the image dimension is increased because of 2D-Upsampling, while the number of feature channels is reduced during each up-sampling. Moreover, we used a channel-wise attention block (marked in green) to concatenate the input image with the output of decoder parallel block. In the channel-wise attention block, global max-pooling was used to compute the feature maps from the output of decoder parallel block and passed to the dense layer to generate attention weight using the sigmoid activation function. Then the attention weight is multiplied with the output of the decoder parallel block and finally concatenated with the input. In the final stage, a 1 × 1 2D-convolution layer with C = 1 is applied to predict the class of each pixel, followed by a sigmoid activation function, where C is the class for binary segmentation.

**Figure 3:**
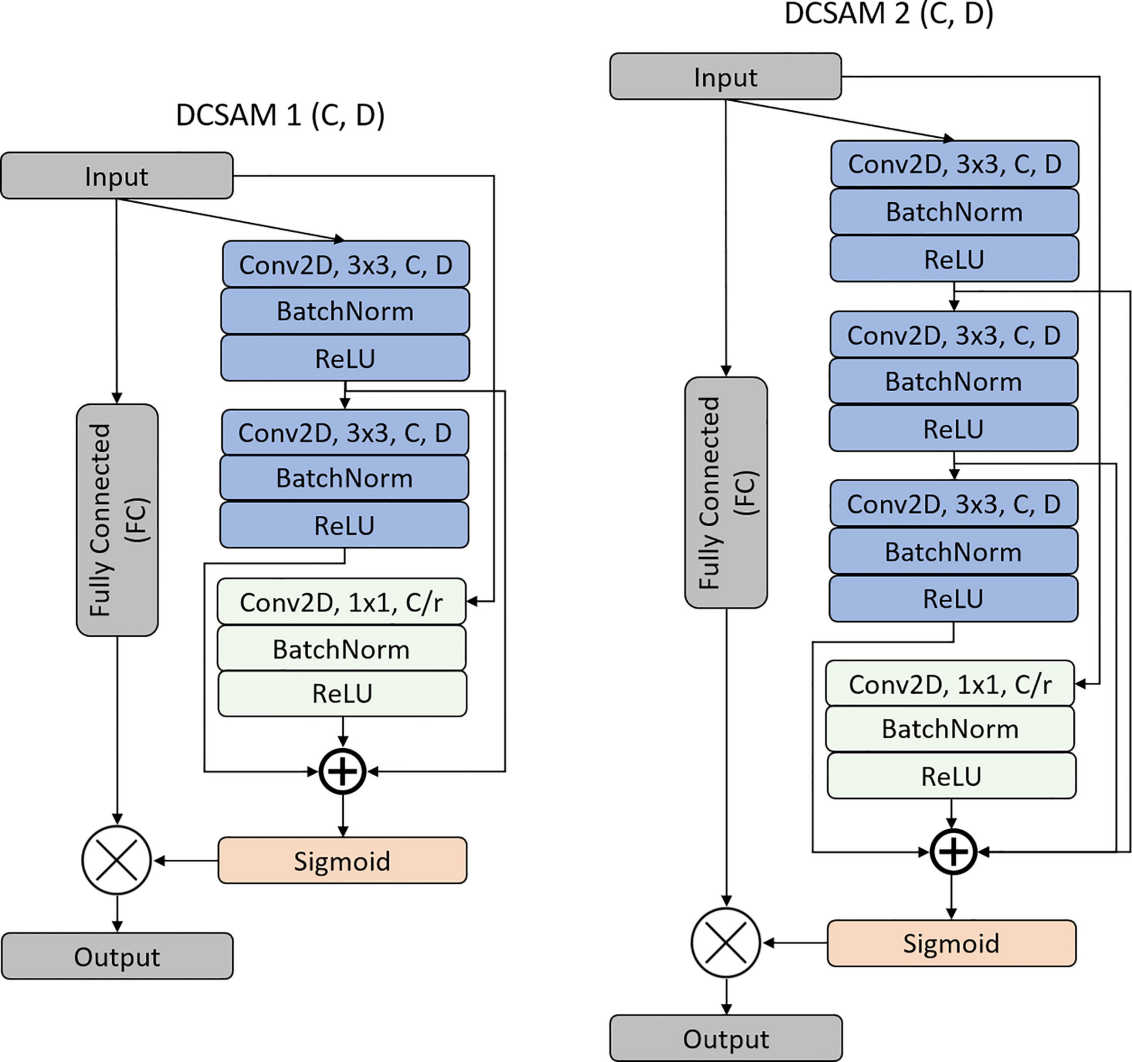
Structure of the Densely Convolutional Spatial Attention Module (DCSAM).

During image segmentation, “attention” refers to a strategy of highlighting just the relevant activations. This saves computing resources by lowering the number of irrelevant activations, allowing the network to generalize more effectively. In essence, the network pays attention to a specific area of the image. Soft attention works by assigning varying weights to different parts of the image. High-importance areas are given a greater weight, whereas low-importance parts are given a lower weight. As the model is trained, the weighted regions receive more attention. Inspired by the work of attention (43, 45, 46), we propose a DCSA module (DCSAM), which is used in the encoder and decoder part of the network, shown in **Figure 3**. However, this module is designed simply by stacking convolutional blocks with 1 × 1 and 3 × 3 filters followed by soft attention.

#### 3.4.1 Densely Convolutional Spatial Attention Module

The DCSAM introduces a building block for our proposed CNN model that improves channel interdependencies. This module consists of regular convolution (i.e., kernel size = 3 × 3) and squeeze blocks (i.e., kernel size = 1 × 1) with different parameters and fully connected (FC) layers. However, we produce a very simple spatial attention module (SAM) based on three different techniques: 1) The input feature map $\text{F}\in {\mathbb{R}}^{\text{H}\times \text{W}\times \text{C}}$ is passed through 3 × 3 and 1 × 1 convolutional layers, separately. 2) The dilation rate (D) is used during the down-sampling convolution operations to increase the effective receptive field of the network. 3) Finally, $\text{F}\in {\mathbb{R}}^{\text{H}\times \text{W}\times \text{C}}$ is passed separately through the FC layer with the ReLU activation function to multiply the dense features, and the concatenated output of the convolutional blocks is passed through another FC layer with the Sigmoid activation function. To save the parameter, a 1 × 1 attention convolutional layer is processed by reducing the channel dimensions to ${\mathbb{R}}^{\text{C}/\text{r}\times 1\times 1}$ ), where r is the reduction ratio (e.g., if 256 is the channel vector and r is 8, then the number of neurons in the hidden layer is 32). In short, the DCSAM-1 can be computed as follows:


$$\text{conv}1=\text{R}\left[\text{BN}\left(f^{3\times 3}\left(\text{Conv}\left({\text{F}}_{Map}^{In}\right),\text{ D}\right)\right)\right]$$

$$\text{conv}2=\text{R}\left[\text{BN}\left(f^{3\times 3}\left(\text{Conv}\left(\text{conv}1\right),\text{ D}\right)\right)\right]$$

$$\text{squeeze }=\text{R}\left[\text{BN}\left(f^{1\times 1}\left(\text{Conv}\left({\text{F}}_{Map}^{In}\right)\right)\right)\right]$$

$$\text{concatenate}=\left[{\text{F}}_{Map}^{In}\;⊕\;\text{conv}1\;⊕\;\text{conv}2\;⊕\;\text{squeeze }\right]$$

$$\text{fully connected}1=\text{R}\left[\left(\text{FC}\left({\text{F}}_{Map}^{In}\right)\right)\right]$$

fully connected2=σ[FC(concatenate)]

(1) $${\text{F}}_{Map}^{Out}=\text{fully connected}1⊗\text{fully connected}2$$

where ${\text{F}}_{Map}^{In}$ is the input; Conv is the convolution; $f^{3\times 3}$ and $f^{1\times 1}$ are the filter kernel sizes of the convolution blocks; BN is Batch Normalization; R and σ are the ReLU and Sigmoid activation functions, respectively; ⊕ and ⊗ denote the concatenation and elementwise multiplication functions, respectively; and ${\text{F}}_{Map}^{Out}$ is the output of the DCSAM.

### 3.5 Training details

The model training was performed with patch images of size 128 × 128 pixels obtained from the original extracted images of size 512 × 512 pixels to reduce the computational cost and to achieve accurate segmentation results. All networks were trained for 100 epochs, and the ReduceLROnPlateau function was used to monitor the loss of validation during training. A factor of 0.8 and patience of 5 were set; thus, if there was no improvement in validation loss for five consecutive epochs, the learning rate was reduced by a factor of 0.8. The training data were shuffled at the beginning of each epoch, and the batch size was set to 8. We used the Adam optimizer (52) to change the attributes of the neural network, such as weights and learning rate, to reduce loss and solve optimization problems by minimizing the objective function. The training duration was approximately 240 min on an NVIDIA RTX 3060 GPU. The experiments were performed on a Windows 10 operating system, and the model was implemented with the Tensorflow DL framework.

### 3.6 Machine-learning approach

Nuclei segmentation was also performed based on the traditional machine-learning approach, in which we used seven different types of filters (i.e., Gabor, Canny Edge, Sobel, Scharr, Robert, Prewitt, Gaussian, Median) (53–56) to extract the meaningful features from the annotation area of the cell nuclei images for semantic segmentation using a random forest (RF) classifier (57, 58). These filters extract texture- and edge-based features, which are useful for the suitable segmentation of images because of optimal localization properties covering both the spatial and frequency domains (56). We used an RF algorithm for nuclei segmentation, as it is capable of handling complex problems by ensembling multiple classifiers together and it performs well compared to other methods in terms of overfitting and precision.

### 3.7 Statistical evaluation

The performance was evaluated with quality measures commonly used in segmentation tasks to calculate the similarity between the ground truth and model prediction. “Metric” refers to a semantic division of binary values in which nuclei are considered to be in the foreground and everything else is background. For our quantitative study, we used three evaluation metrics, namely, accuracy, Dice coefficient (DC) (59), and Jaccard coefficient (JC) (60). Of these, DC and JC are the most important metrics which provide a similarity measure between the segmentation results and ground truth combining both object- and pixel-level performance. The loss functions used during the training of the models were binary cross-entropy loss, Dice loss, and Jaccard loss. Thus Dice loss and Jaccard loss can be used by computing $1-Dice_{score}$ and $1-Jaccard_{score}$ , respectively.


(2) $$Accuracy_{score}=\frac{\text{TP}+\text{TN}}{\text{TP}+\text{TN}+\text{FP}+\text{FN}}$$

(3) $$Dice_{score}=\frac{2\sum_{i}^{N}\left[M_{w}\left(X_{i}\right)*Y_{i}\right]}{\sum_{i}^{N}\left[M_{w}\left(X_{i}\right)+Y_{i}\right]}$$

(4) $$Jaccard_{score}=\frac{\sum_{i}^{N}\left[Y_{i}*M_{w}\left(X_{i}\right)\right]}{\sum_{i}^{N}\left[Y_{i}+M_{w}\left(X_{i}\right)–Y_{i}*M_{w}\left(X_{i}\right)\right]}$$

(5) $$Binary_{loss}=–\frac{1}{N}\sum_{i=1}^{N}\left[Y_{i}*\log\left(M_{w}\left(X_{i}\right)\right)+\left(1–Y_{i}\right)*\log\left(1–M_{w}\left(X_{i}\right)\right)\right]$$

where N is the number of output classes, $X_{i}$ is the input sample, $Y_{i}$ is the ground truth of each pixel, $M_{w}$ is the model with network weight ( w ), and $M_{w}$ ( $X_{i}$ ) is the model prediction.

## 4 Ablation experiments

In this section, we show the effectiveness of different models and compared them with our proposed approach. However, to perform this experiment, a total of 3,675 128×128 color images were drawn from RUMC and YUHS datasets (512×512 pixels) which belong to three classes (i.e., GP 3, 4, and 5). Of these, 80% (i.e., 2,940 images) were used for training and 20% (i.e., 735 images) for validation. Therefore, the quality check was carried out for the trained models, especially for parallel CNN blocks (**Figure 2B**) and DCSAM (**Figure 3**) using the validation dataset. As shown in **Supplementary Table S1**, we analyze the trainable parameters, memory usage, validation error, and time per inference step (GPU) to demonstrate the effectiveness and performance of each model. From **Supplementary Table S1**, we can observe that the error percent is reduced when UNet was modified with parallel block and DCSAM. Therefore, we combined both methods to develop our final proposed model which performed well and showed promising results by reducing the validation loss to 3.38% which is much lower than other architectures.

## 5 Experimental results and discussion

To perform the nuclei segmentation, we used data sourced from two different hospitals. The model was evaluated on an independent data set (the data were not used for training) that contained images of PCa with GP 3, 4, and 5. **Table 1** summarizes the experimental results of using the three different metrics (i.e., Eq. 2-4). We used several CNN models, namely U-Net, VGG16-UNet, ResNet50-UNet, DenseNet121-UNet, InceptionV3-UNet, Attention U-Net, and Residual Attention U-Net (61) for nuclei segmentation and compared the results to those from our proposed method using an identical test data set. These CNN models are the standard segmentation architectures used widely by other researchers for various segmentation tasks (62, 63). We also compared the segmentation results with the traditional machine-learning approach using an RF classifier and other tools (i.e., Stardist and ImageJ). The model evaluation was performed on 294 patches extracted from independent six images (three from RUMC and three from YUHS) that were unknown to the trained model. On the other hand, for external validation, we obtained the MoNuSeg dataset and extracted 294 128×128 color images from six multi-organ images, namely liver, breast, kidney, bladder, prostate, and stomach.

**Table 1:** Comparison results on the RUMC and YUHS test datasets.

Value (95% confidence interval)	Average Accuracy (%)	Average Dice Coefficient (%)	Average Jaccard Index (%)
U-Net	95.9 (95.7 – 96.1)	76.8 (75.5 – 78.1)	63.5 (62.0 – 65.1)
Attention U-Net	96.1 (95.9 – 96.4)	78.2 (77.1 – 79.4)	65.5 (64.0 – 66.7)
Residual Attention U-Net	96.2 (96.0 – 96.4)	79.0 (77.9 – 80.2)	66.3 (65.0 – 67.7)
VGG16-UNet	96.2 (96.0 – 96.4)	78.5 (77.3 – 79.7)	65.7 (64.3 – 67.1)
ResNet50-UNet	96.1 (96.0 – 96.4)	77.9 (76.6 – 79.4)	65.2 (63.7 – 66.8)
DenseNet121-UNet	96.1 (95.9 – 96.3)	78.2 (77.1 – 79.2)	64.9 (63.7 – 66.2)
InceptionV3-UNet	95.8 (95.6 – 96.1)	76.5 (75.2 – 77.7)	62.9 (61.5 – 64.3)
Random Forest	95.2 (95.0 – 95.4)	76.8 (75.4 – 78.2)	63.7 (62.2 – 65.2)
StarDist	95.3 (95.1 – 95.4)	81.2 (80.9 – 81.4)	65.5 (63.9 – 67.2)
ImageJ	94.9 (94.8 – 95.0)	73.4 (72.8 – 74.1)	58.3 (57.5 – 59.2)
Proposed Model	96.4 (96.2 – 96.6)	81.8 (80.8 – 83.0)	69.3 (68.2 – 70.0)

The performance metrics for each method were computed based on 294 patches (128 × 128 pixels) extracted from six (i.e., 3 from RUMC and 3 from YUHS) images (512 × 512 pixels).

**Figure 4:**
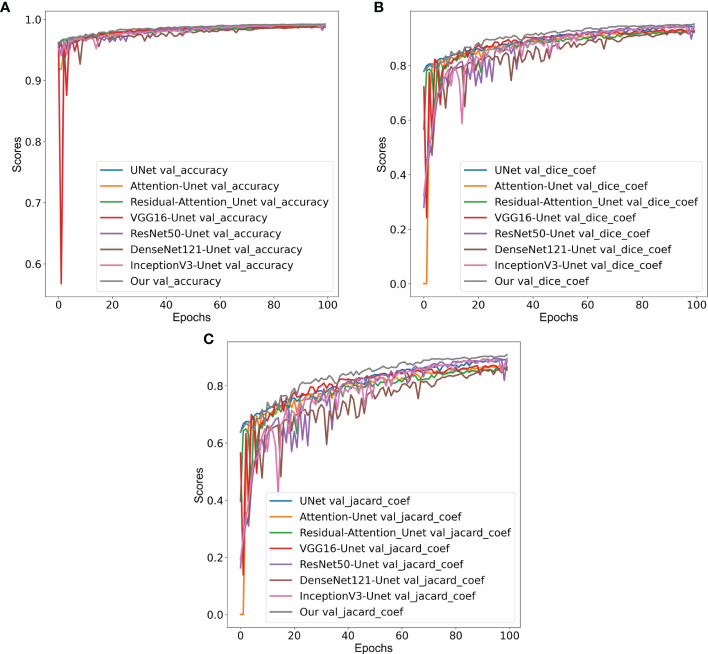
Comparison of validation **(A)** accuracy, **(B)** Dice coefficients, and **(C)** Jaccard coefficients of various convolution neural networks: VGG16-UNet (blue), ResNet50-UNet (orange), DenseNet121-UNet (green), InceptionV3-UNet (red), U-Net (violet), Attention U-Net (brown), Residual Attention U-Net (pink), and our proposed model (gray).

From the comparative analyses (**Table 1**), it is evident that the proposed CNN model DCSA-Net outperformed all others with overall accuracy, DC, and JC of 96.4%, 81.8%, and 69.3%, respectively. Similar to the internal datasets (i.e., RUMC and YUHS), our proposed model performed well and produced promising results for accuracy (87.2%), DC (73.2%), and JC (58.0%) on the MoNuSeg dataset, as shown in **Table 2**. However, for the external dataset, ImageJ achieved the best results with overall DC and JC of 77.1% and 62.9%, respectively. Therefore, from **Tables 1**, **2**, we can say that our designed model is superior compared to the existing methods like U-Net, Attention U-Net, VGG16-UNet, DenseNet121-UNet, InceptionV3-UNet, and Random Forest. **Supplementary Figure S4** shows the learning graph for each model, where we plotted the learning process and compared the accuracy, DC, and JC to analyze the performance based on each metric. From the learning graph (**Figure 4**), it is apparent that our proposed model DCSA-Net (gray plot) achieved the highest accuracy (**Figure 4A**), DC (**Figure 4B**), and JC (**Figure 4C**) values of the validation sets, whereas Attention U-Net (brown plot) achieved the lowest scores. Moreover, our proposed model showed an improvement in accuracy, DC, and JC after only the 22nd epoch and continued improving up to the maximum of 100 epochs.

**Table 2:** Comparison results on MoNuSeg dataset.

Value (95% confidence interval)	Average Accuracy (%)	Average Dice Coefficient (%)	Average Jaccard Index (%)
U-Net	82.1 (81.7 – 82.6)	69.9 (69.0 – 70.9)	54.4 (53.4 – 55.5)
Attention U-Net	86.9 (86.7 – 87.2)	70.6 (69.8 – 71.5)	55.0 (54.1 – 56.1)
Residual Attention U-Net	87.6 (87.3 – 87.9)	73.1 (72.3 – 73.9)	58.0 (57.0 – 59.0)
VGG16-UNet	84.9 (84.6 – 85.2)	64.4 (63.6 – 65.3)	47.8 (47.0 – 48.8)
ResNet50-UNet	86.2 (85.9 – 86.5)	69.2 (68.6 – 69.8)	53.1 (52.4 – 53.9)
DenseNet121-UNet	85.7 (85.4 – 86.0)	67.7 (66.9 – 68.3)	51.3 (50.5 – 52.2)
InceptionV3-UNet	85.9 (85.6 – 86.3)	68.8 (68.4 – 69.3)	52.6 (52.1 – 53.2)
Random Forest	85.5 (84.8 – 85.9)	65.4 (64.0 – 66.8)	50.7 (49.2 – 52.2)
StarDist	83.9 (83.2 – 84.6)	67.5 (64.6 – 67.6)	51.0 (49.4 – 52.6)
ImageJ	87.0 (86.9 – 87.2)	77.1 (76.8 – 77.6)	62.9 (62.4 – 63.5)
Proposed Model	87.2 (86.9 – 87.5)	73.2 (71.3 – 72.9)	58.0 (57.0 – 59.0)

The performance metrics for each method were computed based on 294 patches (128 × 128 pixels) extracted from seven multi-organ (i.e., Liver, Breast, Kidney, Bladder, Prostate, and Stomach) images (512 × 512 pixels).

**Figure 5:**
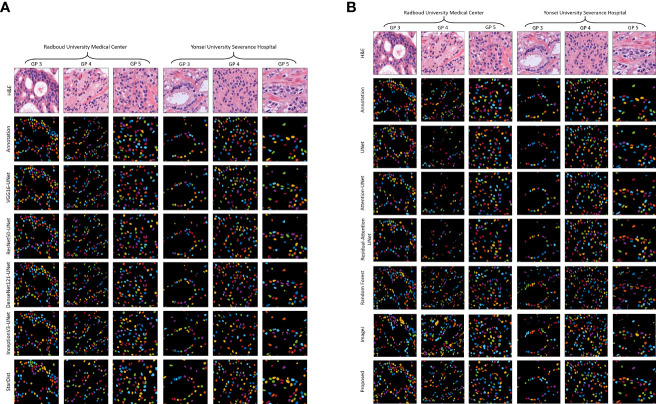
Results of nuclei segmentation based on a variety of artificial intelligence algorithms. Examples of the test, annotation, and predicted images were taken from the center portion of the original images for clear visualization. The annotated samples were used for evaluation. The resulting images of each model are shown in their respective row. **(A)** Results of the pre-trained U-Net. **(B)** Results of the customized U-Net, machine learning algorithm (i.e., Random Forest), and ImageJ. H&E: hematoxylin and eosin.

**Figure 6:**
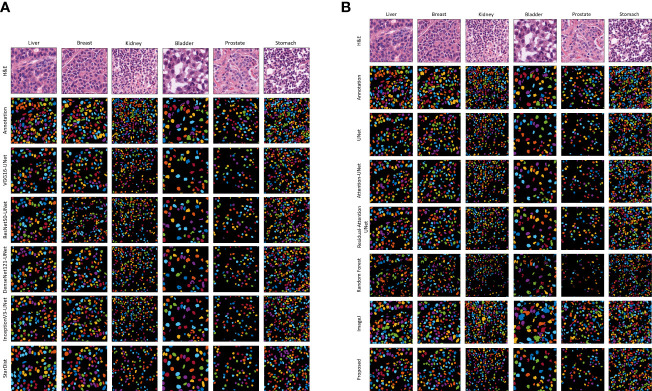
Qualitative segmentation results of different AI models on the external test dataset. The resulting images of each model are shown in their respective row. **(A)** Results of the pre-trained U-Net. **(B)** Results of the customized U-Net and machine learning algorithm (i.e., Random Forest), and ImageJ. H&E: hematoxylin and eosin.

**Figure 5** shows the results of the proposed segmentation model for the internal test dataset that was not included in the training data. We cropped the original, ground truth, and predicted images (512×512 pixels) from the center portion (256×256 pixels) for better visualization of the segmented results. The first and second rows of **Figures 5A, B** show the normalized and annotation samples from which we analyzed how accurately the models segmented the cell nuclei. Rows 3, 4, 5, 6, and 7 of **Figure 5A** show the predicted results of pre-trained VGG16-UNet, ResNet50-UNet, DenseNet121-UNet, InceptionV3-UNet, and StarDist models, respectively. Rows 3, 4, 5, 6, 7, and 8 of **Figure 5B** show the predicted results of U-Net, Attention U-Net, Residual Attention U-Net, RF, ImageJ, and our proposed method, respectively. From row 8 of **Figure 5B**, it is evident that the proposed method outperformed the other models. The nuclei are clearly segmented with high accuracy, according to the annotation samples, and the results are free of noise. On the contrary, we also obtained an external dataset (i.e., MoNuSeg) to perform testing on the trained models and determine their performance for the unknown dataset that contains multi-tissue images. **Figure 6** shows segmentation results for the external dataset. Similar to **Figure 5**, the 512×512 images were cropped from the center portion for better visualization of the results. From row 7 of **Figure 6B**, it is evident that the ImageJ software tool outperformed the other models in terms of DC and JC. However, when comparing the visualization results in **Figures 5**, **6**, it is evident that the StarDist model performed excellently with no background noise and better shape representations compared to other models.

Nuclei segmentation is useful for a variety of biological purposes, including making quantitative assessments of tissue cellular composition. Yet because of variation in shape, inadequate slide digitization, and the presence of overlapping or contact zones, nuclei segmentation is a difficult challenge. In this experiment, we used two distinct datasets with different ethnic origins. We performed stain normalization to standardize the tissue appearance and ensure the robustness of our comparison samples. It should be noted that very poor sample preparation or poor digitization can adversely affect segmentation. However, these problems rarely occur and can be remedied with stricter quality control during tissue preparation and slide scanning. In this study, we introduced a small data set of H&E-stained histopathology images of PCa with more than 16,000 nuclei annotations. Moreover, we enhanced an encoder-decoder CNN architecture to accomplish effective nuclei segmentation. We designed a DCSA network based on U-Net. Our proposed customized model achieved the best and most promising results against an internal test data set, with accuracy, DC and JC scores of 96.4% (95% confidence interval [CI]: 96.2 – 96.6), 81.8% (95% CI: 80.8 – 83.0), and 69.3% (95% CI: 68.2 – 70.0), respectively. Also, the proposed model performed well on an external dataset by giving accuracy, DC, and JC scores of 87.2% (95% CI: 86.9 – 87.5), 73.2% (95% CI: 71.3 – 72.9), and 58.0% (95% CI: 57.0 – 59.0), respectively. However, for the external dataset, ImageJ achieved the highest DC and JC scores of 77.1% (95% CI: 76.8 – 77.6) and 62.9% (95% CI: 62.4 – 63.5), respectively. The reason ImageJ tool achieved better results on the external dataset is that it has been extensively tested and validated over many years and designed to be highly adaptable to a wide range of image analysis tasks, including nuclei segmentation.

Several researchers have developed segmentation models using publicly accessible cell nuclei data sets from multiple organs and performed comparative analyses. However, in this study, we performed the training of CNN models based on single-organ nuclei segmentation and tested with multi-organs data set to analyze the generalizability of the proposed model. StarDist is one of the promising models which performed excellently segmenting the overlapped nuclei compared to other methods thus do not need shape refinement. Nevertheless, the disadvantage is that it can only perform better segmentation with the large size nucleus. Our proposed model can better perform segmentation with high precision than StarDist on small-size nuclei because our training was done with histopathological images of small-size nuclei, whereas, StarDist was trained with larger-size of nuclei. Most CNN networks fail to segment all nuclei, in particular overlapped nuclei, and the watershed algorithm is commonly used in post-processing to partly overcome this weakness. We did not use this technique in our study to separate overlapping nuclei; we only carried out nuclei segmentation and removed the small noisy pixels from the background with an area of less than 30 to improve the overall quality of the segmented image. Moreover, we noticed some image annotation errors in both internal and external datasets, and the inconsistent annotations can introduce noise into the training data which can make it more difficult for the model to accurately segment the images. Therefore, the use of inconsistent ground truth samples with annotation errors for training and validation can leak into the segmentation performance of a DL model. As a pre-processing step, stain normalization improves the performance of nuclei segmentation by reducing variability in the color characteristics of tissue (16, 49, 64, 65). By contrast, many top segmentation techniques depend heavily on data pre-processing, including image smoothing, gamma correction, affine transformations, color deconvolution, and image reconstruction using a generative adversarial network.

## 6 Conclusions

Our method demonstrated superior performance in terms of segmenting nuclei from two different image data sets. It outperformed all standard segmentation algorithms that we tested, and it provided state-of-the-art segmentation of cell nuclei. In the future, we will introduce a large data set of images of prostate and breast cancer tissue from a diverse set of patients. We will perform region-based segmentation of cancerous areas to analyze the tumor-to-stroma ratio of invasive breast cancer. Future work will also involve exploring a segmentation technique based on a convolutional long short-term memory attention network. Although the method reported here is a significant improvement over existing segmentation models, further exploration is needed for better segmentation of touching nuclei using a multi-channel encoder-decoder network based on U-Net. Finally, our experimental results demonstrate that our proposed segmentation technique can achieve better performance through certain improvements to DCSA-Net. Our model can be used in computational pathology for more effective treatment planning *via* a real-time system. Further research will cover nuclei segmentation using both large and small-size nuclei from multiscale images for training purposes to perform optimal segmentation of diverse sizes of the nucleus.

## Data availability statement

The original contributions presented in the study are included in the article/**Supplementary Material**. Further inquiries can be directed to the corresponding authors. The external dataset analyzed for this study can be found in the Kaggle Repository https://www.kaggle.com/c/prostate-cancergrade-assessment. The internal dataset analyzed for this study can be found at : https://figshare.com/articles/dataset/Training_Data_zip/22249291. The code is available at: https://github.com/subrata001/DCSA-Net.

## Ethics statement

The written informed consent of the two hospitals was waved for their participation in the study, which was approved by the Institutional Review Boards of RUMC and YUHS.

## Author contributions

RI: Writing – the code and original draft, analysis and editing, data curation. SB: Conceptualization, Methodology, Writing – the code, original draft, review & editing, data curation. Y-BH: formal analysis, validation. HR: Data curation, validation. H-CK: Formal analysis, Supervision. W-SR: Writing – review. DK: Writing – review. H-KC: Funding acquisition, Project administration, Supervision. N-HC: Data curation, Resources, Writing – review. All authors contributed to the article and approved the submitted version.
