# Supplementary material for: Densely Convolutional Spatial Attention Network for nuclei segmentation of histological images for computational pathology
Source: Front Oncol. 2023 May 25;13:1009681. doi: 10.3389/fonc.2023.1009681 (PMC10248729; doi:10.3389/fonc.2023.1009681)
Supplement: Supplementary file 1 [file DataSheet_1.pdf]

Supplementary Material

1 Supplementary Data

Data sets of PCa histopathology images were sourced from two different centers: Radboud University Medical Center (RUMC) and Yonsei University Severance Hospital (YUHS) to perform nuclei segmentation. Bulten et al. (2022) were the first to use RUMC data set for Gleason grading classification, and they uploaded the data set onto the Kaggle platform, which is publicly available at https://www.kaggle.com/c/prostate-cancer-grade-assessment (accessed on October 10, 2021). The WSIs prepared at RUMC in The Netherlands (Institutional Review Board Approval No. 2016-2275) were scanned with a 3DHistech Panoramic Flash II 250 scanner at ×20 optical magnification. The other data set, from YUHS in Korea (Institutional Review Board Approval No. 1-2018-0044), is not publicly available at this time because of restrictions in the data-sharing agreements approved by the institutional review board. The WSIs in this data set were scanned at ×40 optical magnification with an Aperio AT2 scanner (Leica Biosystems, Vista, CA, USA).

2 Supplementary Figures and Tables

2.1 Supplementary Figures

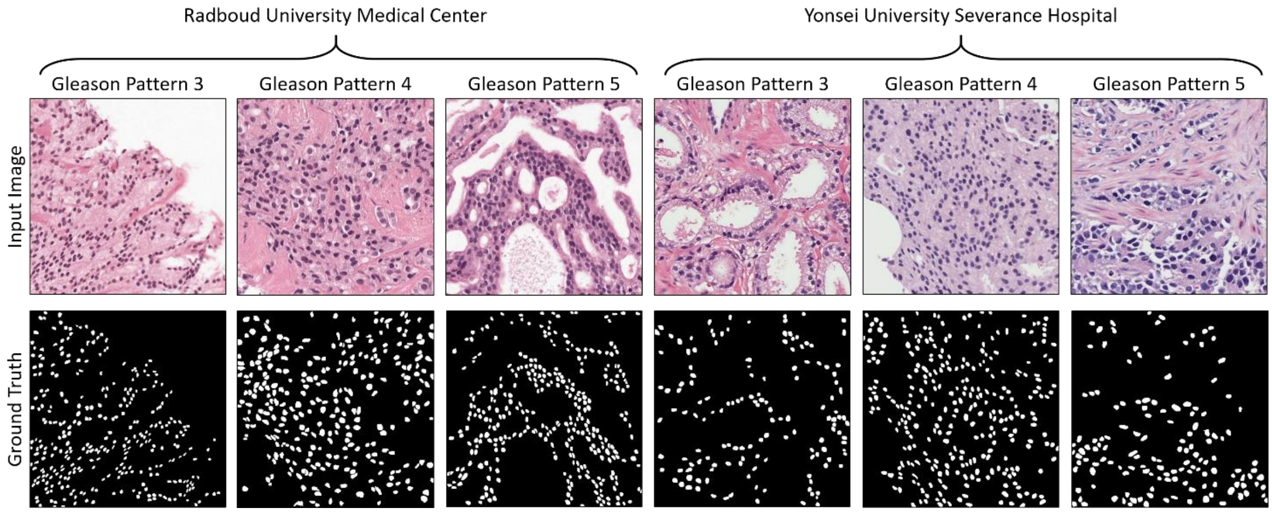

Figure S1. Example of test samples from the RUMC and YUHS datasets.

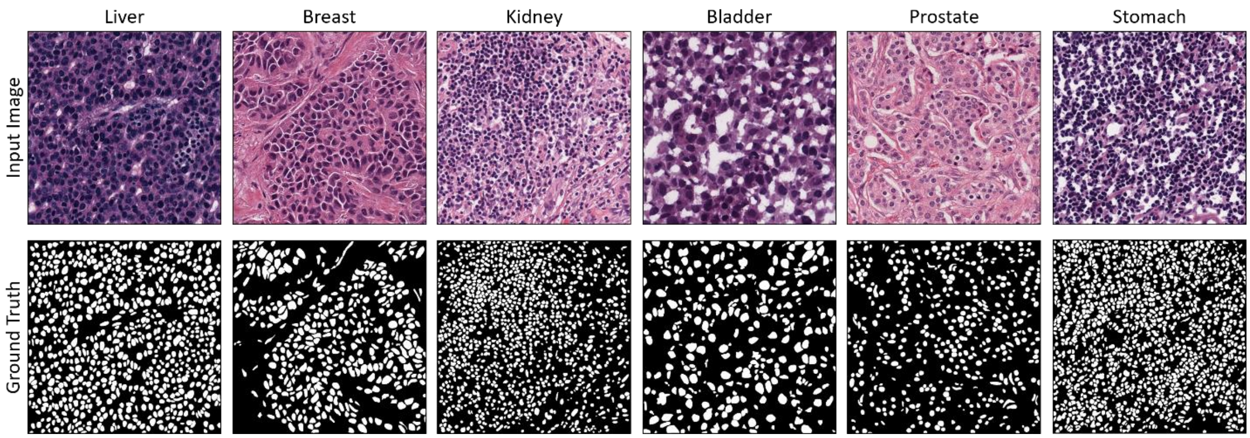

Figure S2. Example of test samples from the MoNuSeg dataset.

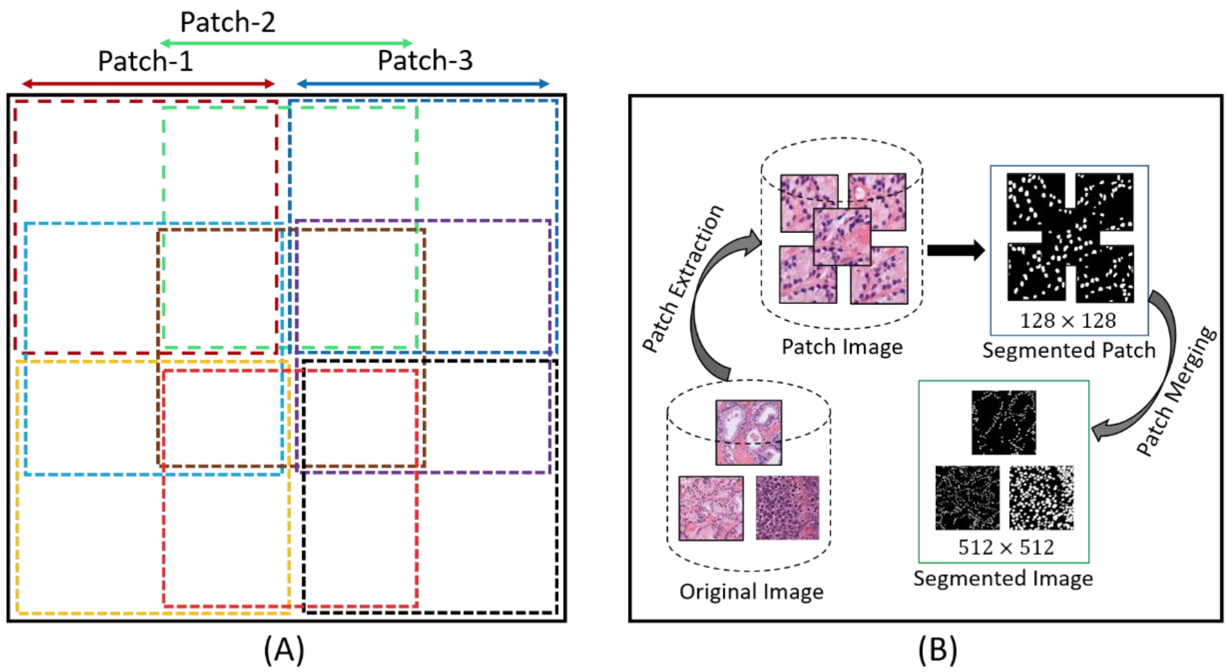

Figure S3. (A) Patch generation pattern. Each color square represents one patch. (B) Patching, segmentation, and reconstruction.

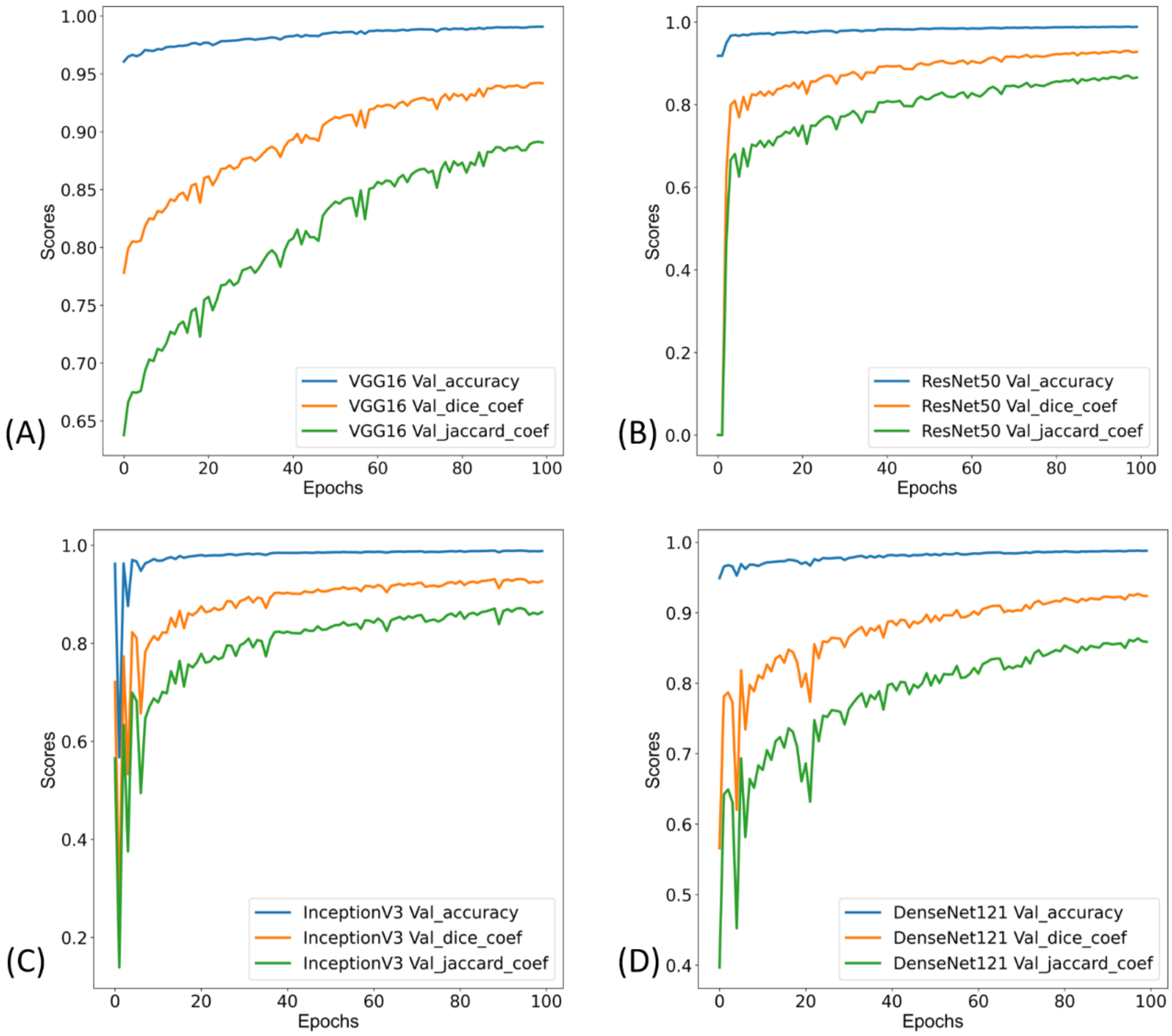
(A)
VGG16 Val_accuracy
VGG16 Val_dice_coef
VGG16 Val_jaccard_coef
Scores
Epochs
(B)
ResNet50 Val_accuracy
ResNet50 Val_dice_coef
ResNet50 Val_jaccard_coef
Scores
Epochs
(C)
InceptionV3 Val_accuracy
InceptionV3 Val_dice_coef
InceptionV3 Val_jaccard_coef
Scores
Epochs
(D)
DenseNet121 Val_accuracy
DenseNet121 Val_dice_coef
DenseNet121 Val_jaccard_coef
Scores
Epochs

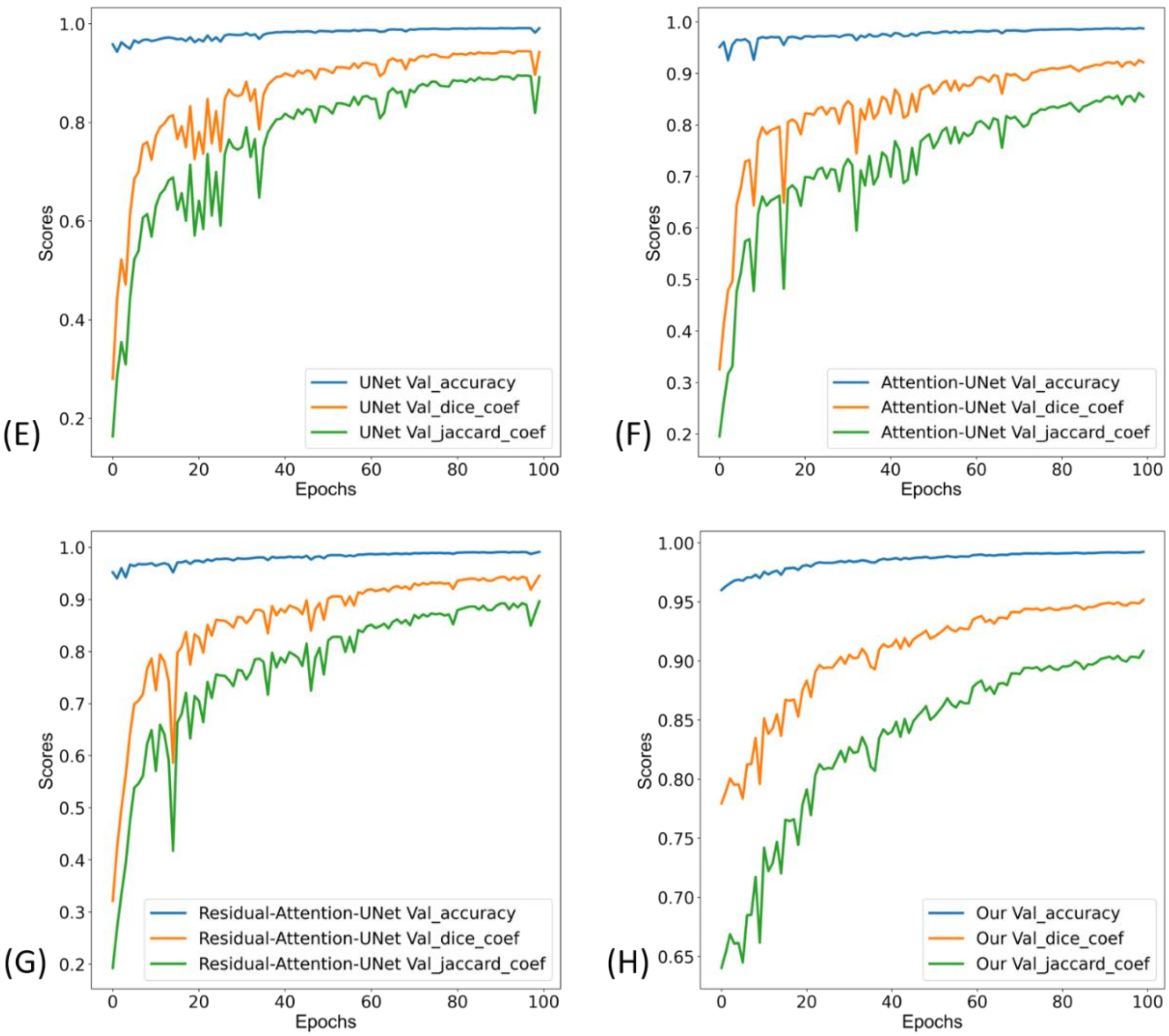

Figure S4. Validation accuracy, Dice coefficient, and Jaccard coefficient of (A) pre-trained VGG16-UNet, (B) pre-trained ResNet50-UNet, (C) pre-trained DenseNet121-UNet, (D) pre-trained InceptionV3-UNet, (E) U-Net, (F) Attention U-Net, (G) Residual Attention U-Net, and (H) our proposed model.

2.2 Supplementary Table

Table S1. Ablation studies to demonstrate the effectiveness of Parallel block and DCSAM in the proposed model.

Architectures	Parameters	Error (%)	Size	Time (sec) per inference step (GPU)
Unet	31.39M	11.55	137.4MB	15.7
Attention_Unet	37.31M	17.75	184.7MB	34.5
ResAttenion_Unet	39.10M	12.65	232.7MB	20.4
model_VGG16	23.74M	31.40	42.0MB	21.0
model_ResNet50	32.51M	34.15	74.3MB	23.3
model_DenseNet121	12.00M	31.15	85.6MB	55.5
model_InceptionV3	29.90M	30.24	48.3MB	41.7
Modified UNet with Parallel Block	31.84M	3.92	159.6MB	19.9
Modified UNet with DCSAM	23.04M	4.34	495.8MB	20.6
Modified UNet with Parallel Block + DCSAM	21.87M	3.38	213.1MB	24.3

3 Reference

Bulten, Wouter, Kimmo Kartasalo, Po-Hsuan Cameron Chen, Peter Ström, Hans Pinckaers, Kunal Nagpal, Yuannan Cai, et al. 2022. “Artificial Intelligence for Diagnosis and Gleason Grading of Prostate Cancer: The PANDA Challenge.” *Nature Medicine* 28 (1): 154–63. https://doi.org/10.1038/s41591-021-01620-2.
